# The Impact of Cereal Grain Composition on the Health and Disease Outcomes

**DOI:** 10.3389/fnut.2022.888974

**Published:** 2022-05-25

**Authors:** Mattia Garutti, Gerardo Nevola, Roberta Mazzeo, Linda Cucciniello, Fabiana Totaro, Carlos Alejandro Bertuzzi, Riccardo Caccialanza, Paolo Pedrazzoli, Fabio Puglisi

**Affiliations:** ^1^Department of Medical Oncology - CRO Aviano, National Cancer Institute, IRCCS, Aviano, Italy; ^2^Department of Anaesthesia and Intensive Care - CRO Aviano, National Cancer Institute, IRCCS, Aviano, Italy; ^3^Department of Medicine (DAME), University of Udine, Udine, Italy; ^4^Clinical Nutrition and Dietetics Unit, Fondazione IRCCS Policlinico San Matteo, Pavia, Italy; ^5^Department of Internal Medicine and Medical Therapy, University of Pavia, Pavia, Italy; ^6^Medical Oncology Unit, Fondazione IRCCS Policlinico San Matteo, Pavia, Italy

**Keywords:** cereals, grains, fibers, cancer, cardiovascular diseases

## Abstract

Whole grains are a pivotal food category for the human diet and represent an invaluable source of carbohydrates, proteins, fibers, phytocompunds, minerals, and vitamins. Many studies have shown that the consumption of whole grains is linked to a reduced risk of cancer, cardiovascular diseases, and type 2 diabetes and other chronic diseases. However, several of their positive health effects seem to disappear when grains are consumed in the refined form. Herein we review the available literature on whole grains with a focus on molecular composition and health benefits on many chronic diseases with the aim to offer an updated and pragmatic reference for physicians and nutrition professionals.

## Introduction

Grains are a vital food category for many populations of the world and their annual production exceeds 2,700 tons with an alignment of supply and demand (1).

Grains represent an important source of carbohydrates, proteins, fibers, minerals, vitamins, and phytochemicals and their regular consumption appears to be associated with many health benefits (2, 3). Indeed, incidence and mortality of several chronic non-communicable conditions like cancers, type 2 diabetes, and cardiovascular diseases have been shown to be reduced in people regularly eating whole grains (3). However, these health benefits might not be replicated by the consumption of refined grains (RG) which are characterized by lower levels of minerals, vitamins, fibers, and phytochemicals (4). This represents an important topic of public health since in the canonical western diet the vast majority of consumed grains are generally in the refined form (5).

Herein we review the available literature on whole grains (WG) with a focus on their molecular composition and their health benefits with the aim to offer an updated and pragmatic reference for physicians and nutrition professionals.

## Composition of Cereal Grains

**Figure 1:**
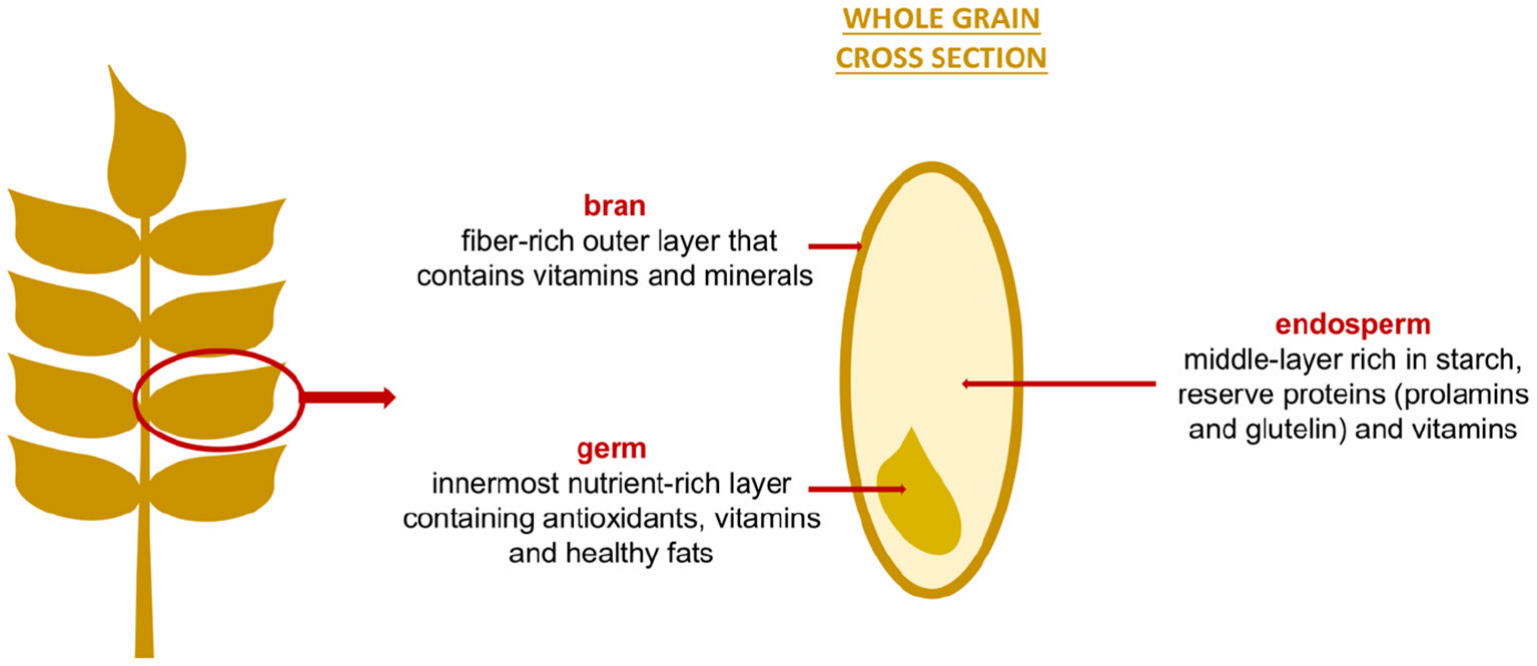
Structure and composition of grains.

Cereals are made of carbohydrates for 50–80% of their weight and contain a lower but significant amount of proteins (5–6%), and lipids (1–10%) (Table 1; Figure 1). Whole grains are an important source of mineral salts (1.5–2.5%) (phosphorus, calcium, magnesium, potassium, iron, zinc, copper), and vitamins (thiamine, riboflavin, niacin, pyridoxine, biotin, folic acid, vitamin E, and vitamin A) (25, 26).

**Table 1:** Nutrients of cereals and pseudocereals.

**Cereals**	**Kcal**	**CHO**	**Pro**	**Fat**	**F_**TOT**_**	**F_**SOL**_**	**Vitamins**	**Micros**	**Phytochemicals**	**Antinutrients**
Barley (*Hordeum vulgare*)	354	73.5	12.5	2.3	17.3	/	Thiamine 0.646 mg	Manganese 1.94 mg	Fibers^U^, anthocyanins (6), polyphenols (7)	Tannins (Proanthocyanidins) (8), phytate (9, 10), oxalates (9)
							Riboflavin 0.285 mg	Zinc 2.77 mg		
							Niacin 4.6 mg	Copper 0.498 mg		
							Pantothenic acid 0.282 mg	Iron 3.6 mg		
							Vitamin B6 0.318 mg	Phosphorus 264 mg		
							Vitamin A 0.007 mg	Magnesium 133 mg		
								Calcium 33 mg		
								Potassium 452 mg		
								Sodium 12 mg		
Buckwheat (*Fagopyrum esculentum Moench*)	343	71.5	13.2	3.4	10	/	Thiamine 0.6 mg	Iron 2 mg	Fibers^U^	Tannins (Proanthocyanidins) (8)
							Niacin 4.4 mg	Phosphorus 330 mg		
								Calcium 67 mg		
								Potassium 311 mg		
								Sodium 1 mg		
								Potassium 450 mg		
								Calcium 110 mg		
								Iron 4 mg		
Emmer (*Triticum dicoccum, Triticum spelta*)	353	69.3	14.6	2.4	6.5	0.96	Niacin 8.511 mg	Phosphorus 387 mg	Polyphenols (11, 12), fibers^U^	NA
								Magnesium 128 mg		
								Calcium 35 mg		
								Potassium 407 mg		
								Zinc 4.79 mg		
								Copper 0.39 mg		
								Manganese 2 mg		
								Iron 1.53 mg		
										
Einkorn (*Triticum monococcum*)	354	62.5	13	2.9	9.8	/	Thiamine 0 mg	Magnesium 125 mg	Polyphenols (11–13), fibers^U^ [β-glucan, lignin, fructans (13)]	NA
							Riboflavin 0.212 mg	Calcium 83 mg		
							Niacin 4.167 mg	Sodium 0 mg		
							Vitamin B6 0.417 mg	Iron 4.5 mg		
							Vitamin A 0.062 mg	Phosphorus 417 mg		
								Zinc 4.69 mg		
								Manganese 3 mg		
Fonio (*Digitaria exilis*)	378	86.67	4.44	1.11	2.2	/		Iron 1.6 mg	Fibers (14)	Phytate (15)
										
Kamut (*Triticum turgidum subsp. turanicum Jakubz*.)	337	70.6	14.5	2.13	11.1	/	Vitamin A 0.003 mg		Fibers^U^	Phytate (9), oxalates (9)
							Thiamine 0.566 mg	Potassium 403 mg		
							Riboflavin 0.184 mg	Sodium 5 mg		
							Vitamin B6 0.259 mg	Zinc 3.68 mg		
							Niacin 6.38 mg	Selenium 0.815mg		
							Pantothenic Acid 0.949 mg	Copper 0.506 mg		
							Vitamin E (alpha-tocopherol) 0.61 mg	Phosphorus 364 mg		
							Vitamin K (phylloquinone) 0.018mg	Magnesium 130 mg		
								Manganese 2.74 mg		
								Iron 3.77 mg		
								Calcium 22 mg		
Maize (*Zea mays* L.)	357	75.1	9.2	3.8	2	/	Thiamine 0.36 mg	Phosphorus 256 mg	Fibers^U^ (fructans, cellulose, β-glucan, arabinoxylan, lignin) (16), phenolic acid (16), anthocyanins (6), polyphenols (7)	Phytate (10, 17), polyphenol (17)
							Riboflavin 0.2 mg	Magnesium 120 mg		
							Niacin 1.5 mg	Calcium 15 mg		
							Vitamin A 0.062 mg	Iron 2.4 mg		
								Zinc 2.21 mg		
								Potassium 287 mg		
								Sodium 35 mg		
								Selenium 0.155 mg		
								Copper 0.31 mg		
Millets (*Panicum miliaceum* L.)	378	72.8	11	4.22	8.5	/	Thiamine 0.421 mg	Calcium 8 mg	Fibers^U^, anthocyanins (6), polyphenols (7)	Goitrogens (18, 19), tannin (10, 17), phytate (9, 10, 17), oxalates (9)
							Riboflavin 0.29 mg	Potassium 195 mg		
							Niacin 4.72 mg	Sodium 5 mg		
							Pantothenic acid 0.848 mg	Phosphorus 285 mg		
							Vitamin B6 0.384 mg	Iron 3.01 mg		
								Magnesium 114 mg		
								Zinc 1.68 mg		
								Copper 0.75 mg		
								Manganese 1.63 mg		
								Selenium 0.027 mg		
Oats (*Avena sativa*)	389	66.3	16.9	6.9	10.6	/	Thiamine 0.763 mg	Calcium 54 mg	Saponins (20), fibers^U^ (fructans, cellulose, β-glucan, arabinoxylan, lignin), phenolic acid (16, 20), polyphenols (7)	Oxalate (9, 18), Saponins (steroidal avenacosides accumulating in the leaves and triterpenoid avenacins in the rootsand) (21), phytate (9, 10)
							Riboflavin 0.139 mg	Potassium 429 mg		
							Niacin 0.961 mg	Sodium 2 mg		
							Pantothenic acid 1.35 mg	Phosphorus 523 mg		
							Vitamin B6 0.119 mg	Iron 4.72 mg		
								Magnesium 177 mg		
								Zinc 3.97 mg		
								Copper 0.626 mg		
								Manganese 4.92 mg		
Rice (*Oryza sativa* L.)	334	80.4	6.7	0.4	1	0.08	Thiamine 0.11 mg	Calcium 24 mg	Fibers^U^ (fructans, cellulose, β-glucan, arabinoxylan, lignin) (16), phenolic acid (16), anthocyanins (6), polyphenols (7)	Phytate (10, 18), tannins (proanthocyanidins) (8)
							Riboflavin 0.03 mg	Potassium 92 mg		
							Niacin 1.3 mg	Sodium 5 mg		
								Magnesium 20 mg		
								Phosphorus 94 mg		
								Iron 0.8 mg		
								Copper 0.18 mg		
								Zinc 1.3 mg		
								Selenium 0.01 mg		
Rye (*Secale cereale* L.)	338	75.9	10.3	1.63	15.1	/	Thiamine 0.316 mg	Calcium 24 mg	Fibers^U^ (fructans, cellulose, β-glucan, arabinoxylan, lignin) (16), phenolic acid (16), β-carotene^U^, Anthocyanins (6)	Phytate (9, 10), oxalates (9)
							Riboflavin 0.251 mg	Iron 2.63 mg		
							Niacin 4.27 mg	Magnesium 110 mg		
							Pantothenic acid 1.46 mg	Phosphorus 332 mg		
							Vitamin B6 0.294 mg	Potassium 510 mg		
							Vitamin A 0.003 mg	Sodium 2 mg		
							Vitamin E (alpha-tocopherol) 0.85 mg	Zinc 2.65 mg		
							Vitamin K (phylloquinone) 0.059 mg	Copper 0.367 mg		
								Manganese 2.58 mg		
								Selenium		
								0.139 mg		
Sorghum [*Sorghum bicolor* (L.) Moench]	329	72.1	10.6	3.46	6.7	/	Thiamine 0.332 mg	Calcium 13 mg	Fibers^U^, β-carotene^U^, anthocyanins (6), polyphenols (7)	Tannins (proanthocyanidins) (8, 17), phytate (9, 10, 17), oxalates (9)
							Riboflavin 0.096 mg	Iron 3.36 mg		
							Niacin 3.69 mg	Magnesium 165 mg		
							Pantothenic acid 3.67 mg	Phosphorus 289 mg		
							Vitamin B6 0.443 mg	Potassium 363 mg		
							Vitamin E (alpha-tocopherol) 0.5 mg	Sodium 2 mg		
								Zinc 1.67 mg		
								Copper 0.284 mg		
								Manganese 1.6 mg		
								Selenium		
								0.122 mg		
Spelt (*Triticum aestivum* L. subsp. spelta)	338	70.2	14.6	2.43	10.7	/	Vitamin K (phylloquinone) 0.036 mg	Copper 0.511 mg	Fibers^U^	Phytate (9), oxalates (9)
							Vitamin B6 0.23 mg	Calcium 27 mg		
							Vitamin E (alpha-tocopherol) 0.79 mg	Iron 4.44 mg		
							Thiamine 0.364 mg	Magnesium 136 mg		
							Riboflavin 0.113 mg	Manganese 2.98 mg		
							Niacin 6.84 mg	Potassium 388 mg		
							Pantothenic acid 1.07 mg	Sodium 8 mg		
								Phosphorus 401 mg		
								Selenium 0.117 mg		
								Zinc 3.28 mg		
										
Teff^Z^ [*Eragrostis tef (Zuccagni) Trotter*]	367	73.1	13.3	2.38	8	/	Thiamine 0.39 mg	Calcium 180 mg	Fibers^U^, β-carotene^U^	Phytic acid (9), lectins (9), saponins (9), goitrogens (9)
							Riboflavin 0.27 mg	Potassium 427 mg		
							Niacin 3.36 mg	Sodium 12 mg		
							Pantothenic acid 0.942 mg	Phosphorus 429 mg		
							Vitamin B6 0.482 mg	Iron 7.63 mg		
							Vitamin E (alpha-tocopherol) 0.08 mg	Magnesium 184 mg		
							Vitamin A 0.003 mg	Zinc 3.63 mg		
							Vitamin K (phylloquinone) 0.019 mg	Copper 0.81 mg		
								Manganese 9.24 mg		
								Selenium 0.044 mg		
Triticale (*X Triticosecale* spp.)	338	73.1	13.2	1.81	14.6	/	Thiamine 0.378 mg	Calcium 35 mg	Phenolic acids (free and bound) (22), proanthocyanidins (22), lignans (22)	Phytate (23), tannin (23)
							Riboflavin 0.132 mg	Iron 2.59 mg		
							Niacin 2.86 mg	Magnesium 153 mg		
							Pantothenic acid 2.17 mg	Phosphorus 321 mg		
							Vitamin B-6 0.403 mg	Potassium 466 mg		
							Vitamin E (alpha-tocopherol) 0.9 mg	Sodium 2 mg		
								Zinc 2.66 mg		
								Copper 0.559 mg		
								Manganese 4.18 mg		
Wheat *(Triticum Aestivum)*	336	65.2	12.3	2.6	9.7	/	Thiamine 0.42 mg	Calcium 35 mg	Fibers (Fructans, cellulose, β-glucan, arabinoxylan, lignin) (16, 24), phenolic acid (16), anthocyanins (6), polyphenols (7)	Lectins (18), oxalate (9, 18), phytate (9, 10, 18), tannin (17)
							Riboflavin 0.14 mg	Phosphorus 304 mg		
							Niacin 5.4 mg	Iron 3.3 mg		
								Copper 0.31 mg		
								Zinc 3.1 mg		
Wheat (*Triticum durum*)	332	62.5	13.0	2.9	9.8	/	Thiamine 0.43 mg	Calcium 30 mg	Fibers (fructans, cellulose, β-glucan, arabinoxylan, lignin) (16, 24), phenolic acid (16), anthocyanins (6), polyphenols (7)	Lectins (18), oxalate (9, 18), phytate (9, 10, 18), tannin (17)
							Riboflavin 0.15 mg	Phosphorus 330 mg		
							Niacin 5.7 mg	Iron 3.6 mg		
							Vitamin A 0.002 mg	Copper 0.40 mg		
								Zinc 2.9 mg		
								Potassium 494 mg		
								Magnesium 160 mg		
								Selenium 0.038 mg		
Wild rice (*Zizania* spp.)	357	74.9	14.7	1.08	6.02	/	Thiamine 0.115 mg	Calcium 21 mg	Fibers^U^, β-carotene^U^	Phytate (18)
							Riboflavin 0.262 mg	Iron 1.96 mg		
							Niacin 6.73 mg	Magnesium 177 mg		
							Pantothenic acid 1.07 mg	Phosphorus 433 mg		
							Vitamin B-6 0.391 mg	Potassium 427 mg		
							Vitamin E (alpha-tocopherol) 0.82 mg	Sodium 7 mg		
							Vitamin A 0.006 mg	Zinc 5.96 mg		
							Vitamin K (phylloquinone) 0.019 mg	Copper 0.524 mg		
								Manganese 1.33 mg		
								Selenium 0.028 mg		
Amaranth	371	65.25	13.6 6	7.02	6.7		Vitamin C 4.2 mg	Calcium 159 mg	Fibers^U^, β-carotene^U^	Oxalate (18), phytic acid (9), lectins (9), saponins (9), goitrogens (9)
(*Amaranthus* spp.)							Riboflavin 0.2 mg	Copper 0.525 mg		
							Niacin 0.923 mg	Iron 7.61 mg		
							Thiamin 0.116 mg	Magnesium 248 mg		
							Pantothenic acid 1.46 mg	Manganese 3.33 mg		
							Vitamin B6 0.591 mg	Phosphorus 557 mg		
							Vitamin E (alpha-tocopherol) 1.19 mg	Potassium 508 mg		
							Vitamin A 0.001 mg	Sodium 4 mg		
								Zinc 2.87 mg		
								Selenium		
								0.187 mg		
Buckwheat (*Fagopyrum esculentum Moench*)	335	70.6	12.6	3.1	10	/	Riboflavin 0.19mg	Calcium 41 mg	Fibers^U^	Phytic acid (9), lectins (9), saponins (9), goitrogens (9)
							Niacin 6.15 mg	Copper 0.515 mg		
							Thiamin 0.417 mg	Iron 4.06 mg		
							Pantothenic acid 0.44 mg	Magnesium 251 mg		
							Vitamin B6 0.582 mg	Manganese 2.03 mg		
							Vitamin E (alpha-tocopherol) 0.32 mg	Phosphorus 337 mg		
							Vitamin K (phylloquinone) 0.007 mg	Potassium 577 mg		
								Sodium 11 mg		
								Zinc 3.12 mg		
								Selenium		
								0.057 mg		
Quinoa	368	64.2	14.1	6.07	7	/	Thiamine 0.36 mg	Calcium 47 mg	Fibers^U^, β-carotene^U^	Phytate (IP6) (9, 18), saponins (9, 17), lectins (9), goitrogens (9)
(*Chenopodium quinoa*)							Riboflavin 0.318 mg	Iron 4.57 mg		
							Niacin 1.52 mg	Magnesium 197 mg		
							Pantothenic acid 0.772 mg	Phosphorus 457 mg		
							Vitamin B-6 0.487 mg	Potassium 563 mg		
							Vitamin A 0.004 mg	Sodium 5 mg		
							Vitamin E (alpha-tocopherol) 2.44 mg	Zinc 3.1 mg		
							Vitamin K (menaquinone-4) 0.011 mg	Copper 0.59 mg		
								Manganese 2.03 mg		
								Selenium 0.085 mg		

*CHO, carbohydrates; Fat, lipids; F_SOL_, soluble fibers; F_TOT_, total fibers; LA, limiting aminoacid(s); Macros, macronutrients; Micro, micronutrients; NA, not available; Oligos, oligonutrients; Pro, proteins. Nutritional values were founded on USDA Database (https://fdc.nal.usda.gov), CREA Database (https://www.alimentinutrizione.it/sezioni/tabelle-nutrizionali), BDA IEO Database (http://www.bda-ieo.it)*.

Four different parts are present in cereal grains: bran, endosperm, germ and aleurone layer. The endosperm is rich in starch, and reserve proteins (prolamins and glutelin) while the bran, the aleurone layer, and the germ have more proteins with essential amino acids, vitamins, minerals, fibers, lipids (greater presence in the germ), and bioactive substances (e.g., phenolic acids, flavonoids, alkylresorcinols, avenantramides, tannins, carotenoids, lignans and phytosterols) (25, 26).

Whole grains represent a widely consumed food in many diets and have been linked with healthy effects (see below). These beneficial effects seem to be, at least in part, attributable to the presence of dietary fibers which overall amount and types varies between different cereals. In general, whole grains and pseudo-cereals are rich in both soluble and insoluble fiber; the most abundant ones are: cellulose, arabinoxylan-glucan, xyloglucan, and fructan (27).

Many vitamins are present in cereals: thiamine, riboflavin, niacin, pyridoxine, biotin, folic acid, pantothenic acid, vitamins A, E, and K (28). Since they are especially distributed in the integument and in the germ, the refinement process can remove a significant amount (29).

In addition, the absolute abundance of vitamins and minerals is determined by a myriad of factors, including cultivar (30), soil composition (30), and the degree of refinement (29).

## Relevant Phytochemicals in Cereals and Their Effect on Health

In cereals, as other plant food, an equilibrium exists between phytochemicals and antinutrients, which could be two aspects of the same compound. Phytochemicals are bioactive molecules characteristic of plants, and they are crucial for the human health by playing a pleiotropic action. Phytochemicals act as anti-oxidants or might have a role in maintaining DNA repair, controlling cell proliferation, cell differentiation, cancer cells apoptosis and DNA metabolism (16, 20). Examples of phytochemicals in cereal grains include terpenoids, polyphenols, phenolic constituents, alkaloids, carotenoids, phytosterols, saponins, and fibers (20, 24) (Table 2).

**Table 2:** Phytochemicals in cereals and their effects on health.

**Phytochemical/antinutrient**	**Potential effect on health**	**References**
Flavonoids, isoflavonoids, anthocyanidins	Benefits: anti-free radicals' action in cellular signaling, cancer prevention	(7, 20, 31–35)
	Harms: platelet aggregation, allergies, inflammation, and hepatotoxins	
Fibers	Benefits: blood cholesterol reduction, prevention of cardiovascular disease, metabolic syndrome, type 2 diabetes and cancer	(16, 20, 24, 36)
Lectins	Benefits: possible cancer diagnostic and treatment tools	(9, 18, 37–42)
	Harms: altered gut function, inflammation, food poisoning by lectin-rich foods, if not prepared correctly	
Oxalates	Harms: probable inhibition of calcium absorption and increased calcium kidney stone formation.	(9, 18, 43)
Phytate (IP6)	Benefits: antioxidant, antineoplastic effect, decreasing kidney stone risk, decreasing osteoporosis risk, decreasing dental calculi risk, preventing age-related cardiovascular calcification	(9, 17, 18, 25, 44–52)
	Harms: chelate calcium, iron and zinc, interfering with their absorption	
Goitrogens	Benefits: antineoplastic effect	(9, 18, 19, 53–55)
	Harms: altered thyroid function (hypothyroidism and/or goiter); inhibit iodine uptake	
Phytoestrogens	Benefits: reduced menopausal symptoms, reduced risk of cardiovascular disease, obesity, metabolic syndrome, type 2 diabetes, cognitive disorders, several forms of cancer	(18, 56–64)
Tannins	Benefits: antioxidant and radical scavenging agents, anticarcinogenic, immunomodulatory, anti-diabetic, anti-obesity and cardioprotective agents	(8, 9, 18, 65–70)
	Harms: inhibit iron absorption, negatively impact iron stores.	
Saponins	Harms: alteration of intestinal epithelial integrity, alteration of lipids absorption (including vitamins A and E), putative hemolysis.	(9, 21, 71)
Proteinase's inhibitors (α-amylase inhibitors, trypsin inhibitors and protease inhibitors)	Benefits: prevention of obesity and type 2 diabetes.	(9, 17, 72, 73)
	Harms: delay in growth, reduction of protein digestibility, decreased glucose absorption rate.	
Anthocyanins	Benefits: antioxidant and anti-hypertension activity, cancer and metabolic syndrome prevention, glycemic and bodyweight control, neurological, hepatic and retinal protection, hypolipidemic agent, enhancing immune response and anti-aging agent	(6, 74–88)

Polyphenols represent an heterogeneous group of compounds, constituted by flavonoids and phenolic acids anthocyanins. They have antioxidant and anti-inflammatory activity, but their bio accessibility depends on the type of polyphenol or cereal matrix involved. In the gastrointestinal ambient, polyphenols can potentially change the gut microbiome favorably (7). Anthocyanins are pigments of colored cereals: wheat, rye, millet, barley, rice, maize, and sorghum (6). Several studies, both *in vitro* and *in vivo*, demonstrated that anthocyanins have positive health effects (6, 74): antioxidant activity (75, 76), inhibition of cholesterol absorption (77, 78), reduction of starch digestibility (79), neuro-protection (80), anticancer (81) and antimetastatic activity (82), anti-hypertension effect (83), retinal protection (84), body fat reduction (85), hepatoprotection (86), prevention of metabolic syndrome (31, 87), enhancement of the immune response (82), anti-aging effect (88).

Among the several properties of phytochemicals, the beneficial interaction with the intestinal microbiota represents an open area of research (89). Jayachandran et al. (90) explained as the “fiber gap”, i.e. a diet with low consumption of fibers, could interfere with gut microbiota equilibrium reducing its healthy metabolites. Some of its positive effects on human health are due to the gut microbiota production of short chain fatty acids (SCFAs), such as butyrate, acetate, propionate: these molecules act as anti-inflammatory and anti-oxidant agents, insulin sensitivity modulators, epigenetic modulators (89). SCFAs are the result of fibers degradations by gut microbiota, which contribute to digest soluble fibers, which otherwise would not be metabolized in the bowl. So that, gut microbiota homeostasis is linked to dietary fibers variety, which influences SCFAs production (89, 91, 92). Myhrstad and colleagues analyzed the results of 16 trials conducted to investigating the role of dietary fiber in modulating gut microbiota and human metabolic regulation (92). Despite of heterogeneity in the microbiota analyses, the reviewed studies considered the different types of SCFA-producing bacteria (i.e., *Bifidobacterium, Ruminococcus, Dorea*, ecc.), in particular *Prevotella/Bacteroides* ratio. Authors reported as gut microbiota rich in *Prevotella* spp. could be linked to fibers-enrich diet, but its association with improved metabolic regulation is still unclear. Besides, a fibers-enrich diet was associated with increased presence of SCFA-producing bacteria, however changing in gut microbiota not always corresponds to host metabolic changes.

Antinutrients reduce nutrient bioavailability, sometimes causing some adverse interference. In fact, this heterogenous group has an important role in cereals composition: phytates, lectins, saponins, enzyme inhibitors, tannins, goitrogens, oxalates, and phytoestrogens. Among antinutrients, we could include also mycotoxins, in particular aflatoxins, since cereals contamination represents a relevant concern for human health in several countries (93). Moreover, the plant microbiome composition could also impact on the amount and the types of compounds present in the grains; however, the exact role on health has yet to be elucidated (94).

Phytic acid, also known as phytate or myo-inositol hexaphosphate (IP6), represents an energy and antioxidant agent in seed germination (44). IP6 binds mineral cations (Fe^3+^, Cu^2+^, Zn^2+^, and Ca^2+^) (95), creating insoluble complexes not digestible by human enzymes, with a consequential decreased mineral bioavailability (9, 45). Cereals contain the highest concentrations of phytate, mainly in the outermost layer (18). Despite their antinutrient activity, phytates might be antioxidants chelating excess iron, thereby avoiding the damaging Fenton reactions (46, 47). Phytate could have pleiotropic effect [enhancing immunity, inhibiting inflammatory cascade, decreasing cell proliferation (44, 47), decreasing kidney stone risk (48), osteoporosis risk (25, 49), dental calculi (50), preventing age-related cardiovascular calcification (51, 52)]. However, these observations need to be confirmed by further research.

Lectins, also named hemagglutinins, are a group of carbohydrate/glycoconjugates-binding proteins (96), which reversibly link characteristic carbohydrate portions on cells, getting involved in autoimmune diseases genesis by presenting wrong immune system codes and stimulation of the differentiation of some white blood cells (possibly leading to cancer, but no studies have yet demonstrated lectins to be carcinogenic) (18, 97–99). Due their resistance to host enzyme and bacterial degradation, lectins arrive functionally and immunologically intact into the small intestine (37). In animal models, binding to glycoconjugates and glycan receptors of the enterocytes on their luminal surface, lectins showed to induce alteration of intestinal integrity by compromising nutrient absorption and reducing growth (9, 38–40). However, considering human studies, lectins might have therapeutic effect as nutraceutical agents: their high affinity and specificity to glycans could be used as cancer diagnostic and treatment tools, as adjuvants, together with conventional chemotherapy agents (41, 42).

Saponins, a various family of secondary metabolites produced by oats only among cereals, have a potent antifungal activity (17, 21). Due their ability to bind to cholesterol group in erythrocyte surfaces, saponins could lead to hemolysis *in vitro* (71) and hinder sterol absorption and activity (including vitamins A and E) (17), however saponins bio accessibility is very low and so these interactions could be considered of uncertain significance *in vivo* (100). Furthermore, saponins could be inhibitors of digestive enzymes (e.g., trypsin, glucosidase, amylase, lipase, and chymotrypsin) causing alterations of intestinal epithelial cells integrity (17). However, saponins have a strong hypocholesterolemic effect, in presence of cholesterol (9).

In cereals, compounds with antinutrient activities include α-amylase inhibitors, trypsin inhibitors, and protease inhibitors (17). The inhibition of α-amylase function increases time of carbohydrate absorption (72), while in human diets trypsin inhibitors reduce protein digestion and consequent amino acids availability, leading to decreased growth rate and pancreatic hyperplasia (17), like protease inhibitors (9). Nevertheless, several studies demonstrated that enzyme inhibitors, including alpha-amylase, alpha-glucosidase, and lipase inhibitors, might prevent type 2 diabetes and obesity (73).

Tannins are high molecular weight polyphenol compounds which link with carbohydrates and proteins with intra- and inter-molecular hydrogen bonds, acting as antioxidant, anticarcinogenic, immunomodulatory, and cardioprotective agents (8, 9, 65–69). Although positive effect of antioxidants, tannins could prevent dietary minerals absorption, such as zinc, copper and iron absorption (9, 70, 101). They are present in cereal grains, seeds, legumes, fruits, juices, cocoa beans, tea, wines and nuts, representing one of the most plenty metabolites among secondary plant ones (8).

Goitrogens are a heterogenous compounds group which include foods, environmental toxins and drugs (102). They play a role in thyroid function alteration increasing goiter and other thyroid diseases risk. With mastication and ingestion, myrosinase (enzyme produced by human microflora and activated in damaged plant tissue) converts the goitrongen glucosinolates to several other compounds: nitriles, thiocyanates, sulforaphane and isothiocyanates (53). Glucosinolates could have a role in preventing cancer but also in impairing thyroid function (54, 55). Among cereal, only millet contains a goitrogenic compounds: C-glycosylflavones which inhibit thyroid peroxidase (TPO), as shown in *in-vitro* models (19).

Both plants and mammals produce oxalic acid, or oxalate, in small amounts: plants for several functions (i.e., plant defense, detoxification of heavy metals and calcium regulation) while in mammals it represents a metabolite of ascorbate, hydroxyproline, glyoxylate, and glycine. Oxalate can form soluble (with sodium and potassium) or insoluble (with iron, magnesium, and calcium) salts or esters (18), reducing absorption and probably contributing to calcium oxalate kidney stone formation due to hyperoxaluria (9, 18, 43). Oxalic acid is present in whole grains in smaller amounts than amaranth in which it is more enriched (18). Considering soluble and insoluble oxalate, wheat bran contains more soluble oxalate than whole grains products (44 mg/100 g in whole wheat flour, 113 mg/100 g dry weight vs. 13.8 mg in oats) (103).

Phytoestrogens are polyphenolic compounds derived from plants with peculiar structural analogies to 17-β-estradiol, female main sex hormone (104). They bind to estrogen receptors (ER) with an higher affinities for β receptor rather than α one and a fainter bond than 17-β-estradiol (56). Intestinal microflora converts lignan phytoestrogens, one of the four phenolic phytoestrogens compounds, to the “mammalian lignans,” enterolactone and enterodiol (57, 104). Lignans are extant in a very small amounts (<0.01 mg/100 g) in whole grains, excepted for multigrain bread (105). According to the currently published literature, phytoestrogens could have positive effects on health (i.e., reducing risk of metabolic syndrome, cardiovascular disease, type 2 diabetes, obesity, cognitive disorders, several cancer types, menopausal symptoms). Their role in increasing estrogen-sensitive breast and uterine cancer risk has not been demonstrated, as their role as endocrine disrupts, but last one only in babies and infants because of their underdeveloped digestive tract (18). Petroski and Minich (18) reported how phytoestrogen-rich products could be considered in cancer prevention (i.e., breast, prostate, endometrial, and colorectal cancer).

## Farming Methods and Processing and Their Impact on Grains Constituents

Numerous studies have showed that organic cereals, relative to non-organic crops, contain higher levels of some vitamins (e.g., vitamin C) and minerals (e.g., iron, magnesium and phosphorus), and are also low in nitrates and pesticide residues (106) (Table 3). Organic foods provide higher levels of anthocyanins, flavonoids, and carotenoids (106). Moreover, regular consumption of these foods has been supposed to be associated with reduced risks of several diseases, such as cancer (141). From a general perspective, organic farming has some pros- and some contra-. Among the former, it should be highlighted the greater biodiversity and the better health of agricultural soil (142, 143); among the latter the crop yield remains low per area compared to conventional farming (144–146).

**Table 3:** Effects of processing on cereals nutrients composition.

**Process**	**Effect**	**References**
Organic farming	May increase levels of vitamin C, iron, magnesium, phosphorus, anthocyanins, flavonoids and carotenoids	(107)
Refining	Reduction of fiber, vitamins, minerals, and phytonutrients. Refining increases the glycemic index	(29, 108)
Soaking	Reduction of phytic acid. Reduction of minerals and extractable proteins from the water	(109)
Germination	With germination, depending on the type of cereal, it improves digestion and protein content; increases crude fiber and the amount of sugars. The quantity of vitamins, mineral salts, oxalates, tannins, phytates, flavonoids and phenols could be also modified.	(110–119)
Fermentation	Starch hydrolysis. Increases the bioavailability of minerals (e.g., calcium, phosphorus, and iron). Variable effect on the glycemic index	(120–127)
Cooking	Water-cooking methods facilitates losses of water-soluble vitamins and minerals; the losses amount varied by the cooking method and the duration. Cooking also increases the glycaemic index and, possibly, the antioxidant activity	(29, 128–140)

With the increase of the world population, it is necessary to identify alternative strategies to the use of chemical substances to preserve crops, in particular cereals, from diseases caused by phytopathogens, because these substances negatively alter the beneficial microbiota of the soil with repercussions also on the final consumer (147). On the contrary, biocontrol uses soil and beneficial microbes associated with the plants themselves in order to stimulate the microbial population, including antagonists and pathogens, so as to promote plant growth and avoid the use of synthetic chemicals (148). This mechanism is expressed through: indirect antipathogenesis in which microbes improve the availability of soil nutrients or nitrogen fixation, and direct antipathogenesis, in which microbes fight pathogens by competition, by antibiosis, through production of antipathogenic substances, by stimulating plant defenses through induced systemic resistance, by enhancing the soil microbiota (suppression of the soil), by hyperparasitism that is the use of parasitic microbes of the pathogen, by the insertion of genetic sequences with antipathogenic action on transgenic crops (148). However, how these harvest methods could impact on cereals nutrients contents is an area of active research.

Cooking is another aspect that might impact the vitamin and micronutrients content in grains. For example, boiling causes little losses of riboflavin relative to microwave and pressure cooking (29); in pasta it has been reported that niacin losses are higher than thiamine and riboflavin (128). Folic acid losses were lower after boiling and frying compared to microwave and pressure cooking (129, 130) while vitamin B12 losses after boiling are moderate (131). Among methods that could cause vitamin C loss, pressure cooking appears to induce the maximum loss (132, 133). Interestingly, a study carried out on two varieties of rice showed that boiling increases antioxidant activities and the glycaemic index but does not impact on the phenolic content (134). However, cooking temperature is a major factor the losses of carotenoids (135–140).

Beyond farming and cooking methods, refining (i.e., removing the bran and germ) has a pivotal role in vitamin and micronutrients loss (29, 108). The main cereals subject to refining are wheat, rice, corn, barley, oats, rye, and millet. After bran and germ removal, a refined grains are composed mainly by the endosperm and is therefore rich in starch.

Germination is the process that converts seeds into plants; it takes place in favorable environmental conditions, including the presence of water, oxygen, and suitable temperatures (110). The effect of germination on the nutritional value of grains can vary by the compound considered. For example, an increase in phenolic compounds, flavonoids, crude proteins, and antioxidant was described in buckwheat Instead, germination decreases phytic acid content (111, 112). In corn, germination causes an increase in phenols, fats, crude fibers, and total proteins content (113, 114) while in Finger millet it increases the sugars, the digestion of proteins, tannins, phytates, and starch (115). In sorghum and millet there is an increase in crude fiber, minerals and vitamins, increases the digestibility of proteins, sucrose, glucose and fructose; on the other hand, oxalates, tannins and phytates decrease (116–119).

Fermentation is the digestion of food compounds by bacteria and/or yeast and represents one of the most ancestral methods for food processing and preservation. Fermentation could improve the sensory profile of foods favoring their preservation (120). Through this process is possible to activate enzymes such as α-amylase and maltase which hydrolyze starch and break it down into maltodextrins and simple sugars. During fermentation there is a decrease of total carbohydrates because of their metabolization by microorganisms (121). Minerals have a very low bioavailability as they are bound to polysaccharides and phytates of the cell wall; for example potassium is inaccessible to digestive enzymes as long as it is chelated by phytate molecules (122). Through fermentation it is possible to release these minerals and make them readily bioavailable (123). Furthermore, fermentation increases the bioavailability of calcium, phosphorus and iron probably because of degradation of oxalates and phytates (124). Regarding glycaemic index, fermentation has variable effects and different studies have showed discordant results (125–127).

Soaking is a practice that consist in dipping whole grains in water for a variable amount of time before being cooked. Through soaking the endogenous phytases are activated and a significant part of phytic acid is removed. For example, soaking sorghum flour for 24 h leads to acid phytic reduction up to 20% (109). However, while soaking can reduce the contents of antinutrients, it might also facilitate the loss of minerals and extractable proteins from the water.

Overall, since the various cooking methods could impact on the nutrients composition of grains, consumers should be advised to alternate cooking techniques and methods of preparations.

## The Effect of Consuming Whole Grains on Obesity

Observational and interventional trials have reported an inverse correlation between whole grain-rich diets and obesity parameters, in contrast to refined grains, which present a lower nutritional quality (27, 149–151). There are different mechanisms through which whole grains can help in regulating the body weight, including the fact that they promote satiety, thus leading to a reduced food consumption (152).

A low glycemic index (GI) diet, typical of those containing whole grains, has been shown to have a higher satiating power than a high GI one, regardless of confounding factors like consistency, flavor or percentage of fiber. A low GI diet is characterized by slow digestion and absorption, thus stimulating gastrointestinal receptors that induce satiety (153).

Among the various whole grain components, dietary fibers play a pivotal role in this setting. When ingested, dietary fibers bind water and form a thick agglomerate that can delay gastric emptying and slow down the bowel movements, leading to a reduced glucose absorption. Moreover, dietary fiber can reduce the synthesis of insulin by the pancreas and reduce the risk of hypoglycaemia in the post-absorption setting, thus inducing early satiety and fatty acid catabolism with reduced fat accumulation (154).

Dietary fibers stimulate the synthesis of intestinal hormones, which exert an effect upon satiety and glucose metabolism. For example, cholecystokinin, which is produced in the small intestine, helps in regulating the release of pancreatic hormones and in controlling gastric distention, but it also exerts an effect at the level of the satiety regulation center in the hypothalamus. Finally, the incretins (GIP and GLP-1), which are also produced at the level of the small bowel, stimulate post-prandial insulin synthesis and glucose homeostasis (155).

Moreover, when processed by the gut microbiota, dietary fibers are broken down into short chain fatty acids (SCFAs), which help in controlling body weight by decreasing gastric emptying rate and increasing the satiating effect. In particular, propionic and acetic acid have been found to reduce non-esterified fatty acids plasma concentration, involved in peripheral and hepatic insulin resistance. In addition, propionate may act on glucose and insulin homeostasis and may stimulate GLP-1 release, as suggested in an intervention trial in which a dinner based on non-digestible carbohydrates has been shown to improve glucose response of the subsequent breakfast (156).

Finally, whole grains have been reported to be rich in prebiotics, which can influence gut microbiota composition thus exerting a positive effect over the host body weight control. The gut microbiota has recently emerged among the regulators of body weight. It has been reported that the microbiota of obese mice and human subjects is composed by less Bacteroidetes and more Firmicutes compared to non-obese subjects. The different composition of the bacterial flora may explain the different rate of absorption of ingested foods, thus explaining the role of dysbiosis in increased body weight (157, 158). However, several lines of evidence have shown that microbiota composition could modulate also systemic inflammation, which is an important factor in glycemic resistance, and thermogenesis, which is a contributor of body mass regulation (159).

## Whole Grains and Cancer

Cancer is caused by a complex interaction between environment and genetic background (160). However, it has been estimated that up to 40% of cancers could be avoided by a healthier lifestyle, including diet (161). Although diet should be evaluated as the whole foods that are assumed by a person (i.e., the specific diet pattern), several lines of evidence highlight a protective role of WG on cancers, particularly on colorectal cancer (CC).

For example, the umbrella review of Tieri et al. (3) has shown a convincing risk-reducing effect of WG on CC incidence. This data is aligned with several lines of evidence that support a protective role of WG on CC [e.g., data from 2018 World Cancer Research Fund's (WCRF)]. However, this work has also showed a putative risk-increasing effect of WG on prostate cancer. This observation is not supported by the evidence of WCRF. Possible explanations for this discrepancy might stem from the different definitions of WG in the studies included in the analyzes or the increased prostate cancer screening (i.e., PSA testing) in the population consuming high levels of WG that could be composed by more health-conscious men (3).

In the pivotal work of Reynold et al., it has been shown that a diet high in WG and/or fibers has a significant lowering impact on CC incidence and whole cancer mortality (162). In particular, the protective effect on CC and mortality in terms of relative risk is 0.84 (IC 95% 0.78–0.89) and 0.87 (IC 95% 0.79–0.95) for high fiber diet and 0.87 (IC 95% 0.79–0.96) and 0.84 (IC 95% 0.76–0.92) for high WG consumption. Although this study represents solid evidence to recommend regular consumption of grains in their whole form, it is not able to show how they exert their protective role on cancer incidence/mortality. In particular, it seems that a large part of the positive health effect of WG could be linked to their high fiber contents (162, 163), although a protective role of other components (e.g., phytochemicals, antioxidants, minerals) could not be excluded.

A regular consumption of fibers, estimated to be at least 25–29 g per day (162), seems to protect against cancer with at least three different mechanisms. First, since fibers are chemical substances that cannot be metabolized by the gastrointestinal tract, their presence could accelerate the feces' transit time, reducing the exposition of carcinogens to colonocytes (164–166). Second, fibers can be digested by gut microbiota (i.e., lactic-acid-producing bacteria) which can produce lactic acid and short-chain fatty acids (SFAs) (e.g., butyrate, acetate, propionate) as a consequence of their metabolism (167). It has been shown that butyrate can be used directly by colonocytes as a growth factor and as a nutrient (167). However, it might also exert some epigenetic effects, for example stimulating the acetylation of histones (168). It has been postulated that this epigenetic activity might exert a protective role on the neoplastic degeneration of colonocytes (168). Third, fibers may slow down food digestion and increase satiety thus favoring the loss of weight. Since several lines of evidence link visceral fat with insulin resistance, hyperinsulinemia, pro-inflammatory state, and cancer, fibers could indirectly reduce cancer pathogenesis acting of body composition (169–173).

Although the anticancer role of other nutrients present in WG is still debated, it appears plausible that some phytochemicals could be able to impair malignant cell transformations or progression. For example, phytates might chelate various metals, reducing the probability of oxidative damage of normal cells in presence of oxygen (174). A putative anticancer role has been hypothesized also for saponins (175) and some other phytochemicals (164).

Phytoestrogens are phytochemical compounds present in some cereals, and, in the past, it was postulated that they increase the risk of some cancers because of their pro-estrogenic effect (56). However, several lines of clinical evidence support the idea that these compounds can instead reduce the risk of hormonal cancers such as breast cancers (176). Although this observation could appear puzzling, the cancer protective effect might derive from their competing activity with endogenous estrogens and their preferential signaling through estrogen receptors beta, that have shown to impair cancer cells growth (177).

Comprehensively, several lines of evidence highlight that high WG intake reduces CC incidence and overall cancer mortality; fewer solid conclusions can be drawn for the other cancer types. Although the protective role of WG on cancers appears to be linked with their fibers content, the effect of other phytochemicals cannot be excluded.

## Whole Grains and Cardiovascular Diseases

Healthy dietary regimens exert a protective role against a number of chronic diseases. In particular, whole-grains cereal products and their components, including cereals fibers and bran, have been consistently found to exert a cardioprotective effect (178–180).

Whole grain and cereals fiber consumption reduces the risk of atherosclerosis and coronary artery disease (CAD) progression. Indeed, a comprehensive analysis that evaluated the association of whole grains consumption and atherosclerotic cardiovascular diseases (CVD) showed that an increased consumption of whole grains significantly reduces the risk of occurrence of CVD (181, 182).

The correlation between whole grain intake and ischemic stroke is less clear. An increased consumption of whole grains has been found to reduce, in a non-significant manner, the risk of stroke when compared to a lower intake. Instead, significant data have been reported for individual whole grain components. In particular, cold breakfast cereals and bran consumption lowers the risk of occurrence of ischemic stroke, while regular popcorn consumption enhances this risk. However, since such a correlation was not reported for light and fat-free popcorn, it could be postulated that several compounds, including *trans* fat, butter and sodium, could be responsible for this effect (179).

Whole grains have demonstrated to have pleiotropic effects and the cardioprotective action related to whole grain consumption depends upon several mechanisms (183). Whole grains are composed by bran, germ and endosperm. When whole grains are processed to produce flour, the endosperm (full of carbohydrates) is retained, whereas bran and germ, along with their constituents (fiber, vitamins, minerals and anti-oxidants) are eliminated. The cardioprotective benefit obtained through the consumption of whole grains is particularly attributable to the effects upon the lipid metabolism: whole grains components, including soluble fiber and phytosterols, act upon numerous lipid intermediates, determining a less atherogenic lipid profile. Moreover, whole grains constituents, like phytoestrogens and anti-oxidants, exert a beneficial effect upon the vascular endothelium, thus reducing the risk of endothelial dysfunction, that is another major risk factor for atherosclerosis (178, 183). In the CARDIA study, whole grains were inversely associated with cell-adhesion molecules related to vascular dysfunction and CDV. In particular, adiponectin, which is a peptide released from adipocytes, exerts a cardioprotective activity and lower plasmatic concentrations of adiponectin are commonly observed in obese subjects as well as those with CVD and can induce HTN and endothelial malfunctioning (184, 185).

Whole grains, cereal fibers and bran intakes have also been proven to reduce the level of circulating inflammatory markers and to lower incidence of elevated blood pressure, which are both recognized cardiovascular risk factors (186, 187).

Among the various cardiovascular risk factor, the role of dysbiosis has recently emerged. Gut microbiota dysregulation induces bowel inflammation, with consequent increase in the permeability of the gut membrane and thereafter increased release of bacterial components and metabolites that can lead to an augmented risk for CVD. Dietary modifications increasing the daily consumption of whole grains can represent a feasible opportunity in this setting, aiming at restoring the integrity of the gut barrier and at improving the composition of the intestinal flora. Whole grains are in fact rich in prebiotics, which can positively modify the composition of the intestinal flora with beneficial effects upon the host (188, 189).

## Whole Grains and Diabetes Mellitus Type 2

Following a regular, balanced diet and practicing moderate physical activity in a regular manner are crucial for both the prevention and management of type 2 diabetes mellitus (T2DM).

Whole grains consumption not only allows to prevent T2DM occurrence, but it also helps in controlling T2DM-related factors, such as overweight and obesity, as it has been reported that a diet rich in whole grains determines a better control of the BMI (121, 179–183).

However, conflicting data exist upon this topic. In fact, a meta-analysis of 26 randomized clinical trials (RCTs) reported a non-significant impact upon body weight control in subjects with an elevated BMI when following a diet rich in whole grains, even though whole grain consumption can actually reduce the body fat percentage, when compared to a diet rich in refined grains (190, 191). If individual constituents are considered, instead, a diet rich in rye helps in controlling and, in particular, in lowering the body weight, differently from refined wheat-based diet (192).

Different pathways can be activated by whole grains and their components (especially dietary fiber), determining an effect upon the glycemic control. Dietary fiber, in particular, increases gastric distention and delays intestinal transit time, thus determining early satiety and leading to increased production of molecules active upon the energetic and glycemic balance. Furthermore, the lower energy density of whole grains reduces the energy intake, which leads to decreased body fat and improved insulin sensitivity. Finally, dietary fiber delays nutrient absorption at the intestinal level, reducing insulin demand and stimulating fat absorption, ultimately reducing the fat storage (191).

Whole grains constituents exert anti-oxidant properties and reduce the synthesis of pro-inflammatory molecules, thereby improving insulin sensitivity and preventing the onset and evolution of T2DM (193).

Whole grains are also rich in vitamins and minerals, which help in improving the glycemic control by both regulating insulin-mediated hepatic glucose uptake (vitamin B complex and magnesium) and regulating the oxidative and inflammatory pathways (vitamin E and zinc). T2D comes with a progressive reduction of intracellular zinc levels and increased urinary excretion: zinc supplementation has been proven to exert anti-oxidant properties and lower the synthesis of inflammatory molecules, such as TNF-alpha and IL-1beta (194–197).

In patients with a diagnosis of diabetes mellitus (DM), glycemic control helps in lowering the risk of T2DM-related micro-/macro- vascular complications. An increased intake of fiber (especially soluble fiber) has been associated with a better glycemic control in diabetic patients. With respect to individual constituents, glycated hemoglobin and fasting plasma glucose have been found to be significantly lower in diabetic patients following a diet including regular oat intake, if compared to dietary regimens rich in other cereals. Moreover, oatmeal intake allows for a better glycemic control, with lower post-prandial glucose and improved insulin activity, when compared with a control meal (191, 198–200).

## Whole Grains and Neurodegenerative Diseases

The activation of oxidative and pro-inflammatory pathways, as well as mitochondrial dysfunction, can alter several physiological mechanisms, ultimately leading to different kinds of neurodegenerative disorders. Novel approaches in regulating this mechanisms could be helpful in treating and preventing the onset of these diseases (201, 202).

Whole grains are rich in polyphenols, which are involved in the regulation of several pathways, including the oxidative and inflammatory ones, and they are also involved in the modulation of the host immune response, as suggested by both observational end experimental studies (203–205). Moreover, polyphenols inhibit mitochondrial dysfunction by activating pro-survival molecules, such as Bcl-2 and PERK and by releasing anti-oxidant enzymes, including catalase and superoxide dismutase (206, 207). Polyphenols can help in improving cognitive functioning by blocking the action of acetylcholinesterase and butyrylcholinesterase, thus inducing metal chelation, autophagy regulation and prion elimination (208).

Preclinical data from mice suggest that resveratrol might prevent neuronal loss. SRT501, an oral formulation of resveratrol, stimulates mitochondrial function by activating SIRT1, an NAD+-dependent deacetylase, in autoimmune encephalomyelitis, an animal model of multiple sclerosis (209). Resveratrol is also associated with a neuroprotective effect in 6-hydroxydopamine (6-OHDA)-induced Parkinson's disease rat model, as evidenced by the reduction in DNA condensation and vacuolization of dopaminergic neurons in the substancia nigra. In addition, a lower expression of COX-2 and TNF-α has been shown (210).

Therefore, due to all these beneficial effects, whole grains and their constituents, especially polyphenols, may exert a beneficial, neuroprotective effect, thus representing a feasible therapeutic opportunity in this setting (211).

## Whole Grains and Autoimmune Diseases

As already reported, whole grain consumption can regulate the organism's inflammatory state. Gluten is a proteic complex that can be found in different types of cereals and it is composed by glutenins and gliadins. The gliadins present in wheat gluten can induce celiac disease (CD) in genetically susceptible individuals: it is a chronic inflammatory disorder characterized by inflammation of the intestinal mucosa with increased permeability, small intestinal villous atrophy and consequent malabsorption. A similar histopathological pattern has been observed in other autoimmune diseases and in healthy people (212, 213).

The exposure of mononuclear cells to gliadins determines the expression of inflammatory cytokines in both CD and non-CD patients (214). Enterocytes, in response to the interaction between gliadins and CXCR3 (a chemokine receptor), release zonulin, which can alter the integrity of the plasma membrane. It has been reported that gliadin intake can lead to the release of zonulin in both CD and non-CD patients, in both cases leading to an increased intestinal permeability, with an increased amount of microbial and dietary elements that can reach the periphery and interact with the immune system (215). Actually, this increased intestinal permeability has been postulated to be associated with multiple forms of autoimmune diseases in genetically predisposed individuals (216–218).

Besides gliadin, the lectin wheat germ agglutinin (WGA) plays a synergistic role. In rat small bowel, WGA stimulates monocytes and macrophages to produce pro-inflammatory cytokines, after binding to N-glycolylneuraminic acid, a glycocalyx sialic acid; this, subsequently, affects intestinal permeability (219, 220). Human data are lacking, although high concentrations of antibody to WGA have been found in CD-patients (221).

Thereafter, diets rich in cereals seem to exert a detrimental effect upon the insurgence of autoimmune disease, especially CD.

However, a better understanding of the effects of whole grains in this setting is needed and, in particular, it could be interesting to evaluate what could be the consequences of eliminating the cereals from the diet in terms of inflammatory response and intestinal permeability, not only in healthy individuals, but also in those presenting with autoimmune disorders (222).

## Whole Grains and Other Chronic Diseases

An increased consumption of whole grains and their constituents, like dietary fiber, can determine a positive outcome upon several gastrointestinal disorders.

In particular, dietary fiber, with its intrinsic characteristics, such as viscosity, solubility and fermentability, can regulate the glycemic and lipidic absorption at the bowel level, it can control the bowel movements and determine an alteration of the intestinal flora (166).

There is evidence supporting the role of dietary fiber in controlling and ameliorating IBS symptoms (mostly in forms of the disease which present with constipation), by regulating the texture and the frequency of the stool output (223, 224).

In IBD, dietary fiber consumption is associated with the production of short-chain fatty acids (especially butyrate), which improve the bowel inflammatory status by up- and down-regulating the anti- and pro-inflammatory cytokines, respectively.

Finally, dietary fiber intake lowers the incidence of diverticular disease by rendering the stools more bulky, attenuating the pressure at the level of the large intestine membrane and thus reducing micro-herniation (166).

Patients with chronic kidney disease (CDK) on hemodialysis are recommended not to include whole grains in their diet, given the high amount of phosphorus contained in these foods. In CKD, phosphorus elimination is impaired, determining secondary hyperparathyroidism and inducing calcium deposits within the blood vessel wall, with consequent higher frequency of occurrence of CVD and negative impact upon survival (225).

As said, whole grains constituents determine a protective effect against a number of different pathologies, such as obesity, insulin resistance, T2DM, CVD and cancer (179, 180). These protective effects can be observed in both subjects with CVD, T2DM and elevated BMI and in healthy individuals; furthermore, it has been postulated that these beneficial effects are present also in patients with kidney disease, especially the ones presenting T2DM, CVD and elevated BMI as comorbidities (225). Whole grain consumption in CKD patients could be therefore reconsidered, but further research is needed to better understand their role in this setting.

Low bone mineral density (BMD) depends upon different non-modifiable and modifiable risk factors, including lifestyle factors. Consequently, it has been postulated that the occurrence of serious complications of low BMD, including fractures, can be avoided through lifestyle changes. The role of diet in ameliorating bone health is well established and dietary regimens including whole grains have been found to determine positive effect upon BMD (226, 227).

Cereals are often part of many people's breakfasts. They are usually considered a healthy food due to the misleading messages that praise their properties, not infrequently found in commercial packages. However, it is important to pay attention to the composition of ingredients which can vary between the different products on the market. In particular, it is necessary to favor those richer in fibers and with low carbohydrate content. In addition, it is important to emphasize the theoretical detrimental effect of phytates in wheat bran that may interfere with absorption of calcium from other foods consumed at the same time (228). For example, having breakfast with wheat bran and milk concomitantly might limit the absorption of calcium with a virtual reduction of the preventive effects in terms of bone health. It should be noted that the same interference with calcium absorption seems attenuated in wheat bran present in bread or other foods, where the cereal and therefore the phytates are present in lower concentrations.

## Conclusion

**Figure 2:**
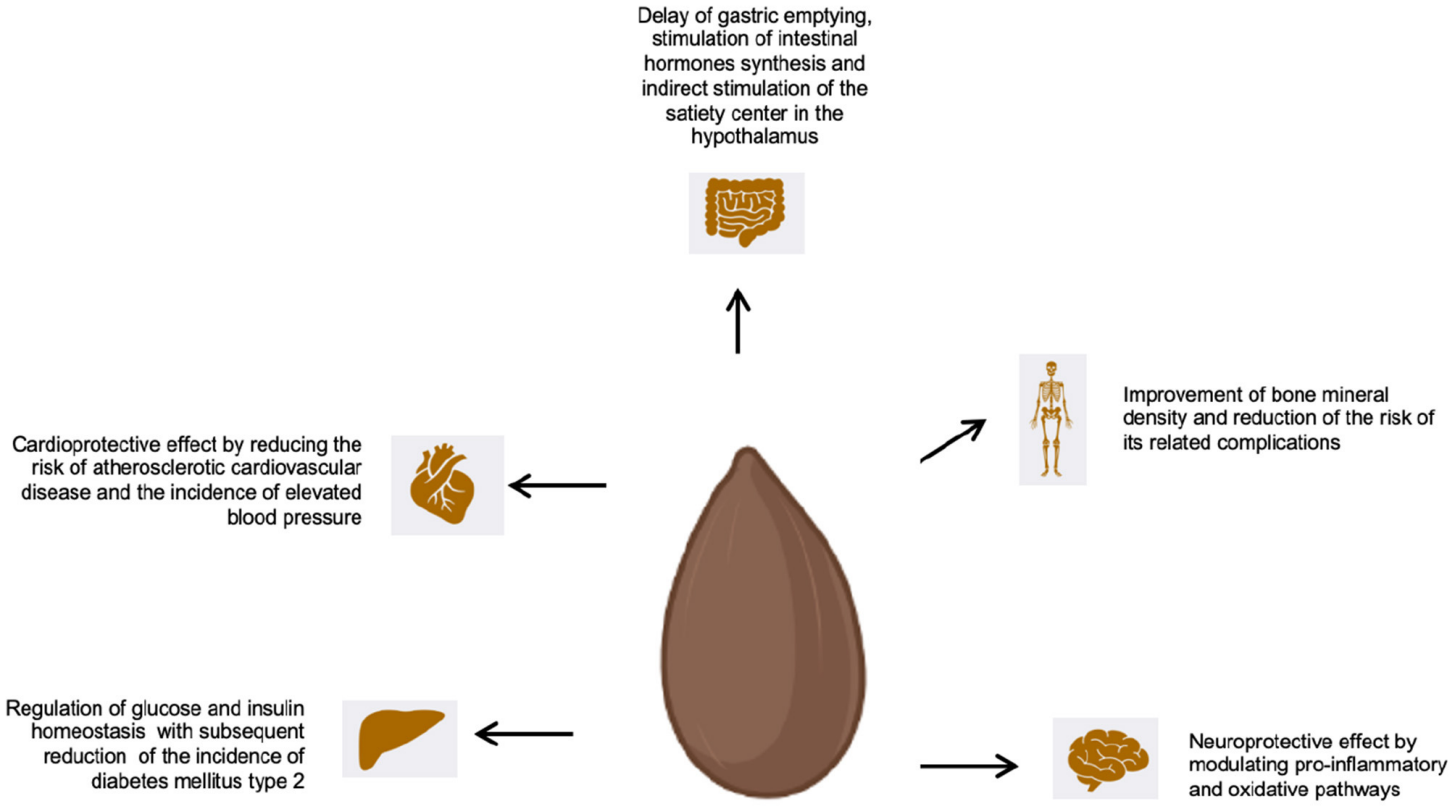
Effect of WG on health and diseases.

Whole grains represent a pivotal food for a healthy diet (Table 4; Figure 2). Besides their carbohydrates content which makes whole grains a main energetic food, whole grains are also an important source of protein, fibers, phytocompounds, minerals, and vitamins. In particular, it should not underestimated their role as source of fibers, since a regular consumption of fibers has shown to be associated with a lower risk of several chronic diseases. Moreover, the recent scientific progress on microbiota field has allowed to appreciate the crosstalk between fibers and commensal flora to impinge on metabolic disfunction and immune dysregulation. Although the complex interplay between fibers/phytocompounds and microbiota has yet to be elucidated, it might represent the crucial pathway behind the healthy effect of whole grains. Moreover, it could be postulated that the identification of microbiota's produced compounds after ingestion of WG could be used to synthetize new drugs or the new generation of supplements.

**Table 4:** Potential effects of whole grains on diseases.

**Disease**	**Potential effects of cereals**	**References**
Cancer	Reduced colon cancer incidence and cancer mortality	(162)
Atherosclerotic cardiovascular disease	Whole grains reduce the risk of atherosclerotic CVDs	(181, 182)
Stroke	Breakfast cereal and bran lower the risk of ischemic stroke; regular popcorn increases the risk of ischemic stroke	(229)
Elevated blood pressure	Whole grains lower the incidence of elevated BP	(187)
Type 2 diabetes mellitus	Whole grains lower the risk of T2DM and related risk factors	(191, 230)
Irritable bowel syndrome	Dietary fiber attenuates IBS symptoms	(223, 224)
Inflammatory bowel disease	Dietary fiber reduces intestinal inflammation	(166)
Diverticular disease	Dietary fiber reduces the risk of diverticular disease	(166)
Chronic kidney disease	Whole grains can exert a beneficial effect in CDK	(225)
Low bone mineral density	Whole grains improve BMD and reduce the risk of its related complications	(226, 227)
Alzheimer's disease	Acetylcholinesterase inhibition by polyphenols, improving synaptic transmission, amyloid β toxicity reduction	(231)
Multiple sclerosis	Neural loss prevention through SIRT1 activation by polyphenols	(232)
Parkinson's disease	Antioxidant and antiapoptotic effect by polyphenols	(211)
Huntington's disease	Genetic and immunological modulation by poliphenols	(233)
Celiac disease	Intestinal mucosal inflammation and increased permeability by gliadin and prolamins	(215)

In addition, it should be underlined the importance of limiting refined grains since they are a poorer source of fibers, phytocompounds, minerals, and vitamins in comparison to their whole counterpart. As stated by the Dietary American guidelines, whole grains should be consumed on a daily basis and at least the half of the quantity should be in the whole form. Substituting refined cereals with their integral counterparts should be facilitated by educational campaigns.

Comprehensively, while the whole grains positive effects on health are clear, in the future it will be crucial to understand the underlying biological mechanisms that govern their activities.

## Author Contributions

MG: conceptualization. MG, GN, RM, LC, and FT: writing and editing. MG and FP: supervision. All authors: methodology and validation. All authors have read and agreed to the published version of the manuscript.

## Funding

This work was supported by the Italian Ministry of Health (Ricerca Corrente) and by contribution 5 × 1,000 per la Ricerca Sanitaria and by Fondazione Costa—Ivrea (TO).

## Conflict of Interest

MG reports advisory board from Novartis, Eli Lilly, Pierre-Fabre, all out-side the submitted work. FP reports receipt of grants/research supports from Astrazeneca, Eisai, Roche, and receipt of honoraria or consultation fees from Amgen, Astrazeneca, Daichii Sankyo, Celgene, Eisai, Eli Lilly, Gilead, GSK, Ipsen, MSD, Novartis, Pierre-Fabre, Pfizer, Roche, Seagen, Takeda, Viatris. The remaining authors declare that the research was conducted in the absence of any commercial or financial relationships that could be construed as a potential conflict of interest.

## Publisher's Note

All claims expressed in this article are solely those of the authors and do not necessarily represent those of their affiliated organizations, or those of the publisher, the editors and the reviewers. Any product that may be evaluated in this article, or claim that may be made by its manufacturer, is not guaranteed or endorsed by the publisher.

## Abbreviations

BMD: bone mineral density
BMI: body mass index
CAD: coronary artery disease
CC: colorectal cancer
CD: celiac disease
CKD: chronic kidney disease
CVD: cardiovascular disease
DM: diabetes mellitus
IBD: inflammatory bowel disease
IBS: irritable bowel syndrome
IP6: myo-inositol hexaphosphate
RCT: randomized clinical trial
SCFAs: short-chain fatty acids
T2DM: type 2 diabetes mellitus
WCRF: World Cancer Research Fund's
WG: whole grains.
